# The International Mouse Phenotyping Consortium: comprehensive knockout phenotyping underpinning the study of human disease

**DOI:** 10.1093/nar/gkac972

**Published:** 2022-10-28

**Authors:** Tudor Groza, Federico Lopez Gomez, Hamed Haseli Mashhadi, Violeta Muñoz-Fuentes, Osman Gunes, Robert Wilson, Pilar Cacheiro, Anthony Frost, Piia Keskivali-Bond, Bora Vardal, Aaron McCoy, Tsz Kwan Cheng, Luis Santos, Sara Wells, Damian Smedley, Ann-Marie Mallon, Helen Parkinson

**Affiliations:** European Bioinformatics Institute, European Molecular Biology Laboratory, Welcome Genome Campus, Hinxton CB10 1SD, UK; European Bioinformatics Institute, European Molecular Biology Laboratory, Welcome Genome Campus, Hinxton CB10 1SD, UK; European Bioinformatics Institute, European Molecular Biology Laboratory, Welcome Genome Campus, Hinxton CB10 1SD, UK; European Bioinformatics Institute, European Molecular Biology Laboratory, Welcome Genome Campus, Hinxton CB10 1SD, UK; European Bioinformatics Institute, European Molecular Biology Laboratory, Welcome Genome Campus, Hinxton CB10 1SD, UK; European Bioinformatics Institute, European Molecular Biology Laboratory, Welcome Genome Campus, Hinxton CB10 1SD, UK; William Harvey Research Institute, Queen Mary University of London, London EC1M 6BQ, UK; Mary Lyon Centre at MRC Harwell, Harwell Campus OX11 7UE, UK; Mary Lyon Centre at MRC Harwell, Harwell Campus OX11 7UE, UK; Mary Lyon Centre at MRC Harwell, Harwell Campus OX11 7UE, UK; Mary Lyon Centre at MRC Harwell, Harwell Campus OX11 7UE, UK; Mary Lyon Centre at MRC Harwell, Harwell Campus OX11 7UE, UK; Research Data Team, The Turing Institute, 96 Euston Rd, London NW1 2DB, UK; Mary Lyon Centre at MRC Harwell, Harwell Campus OX11 7UE, UK; William Harvey Research Institute, Queen Mary University of London, London EC1M 6BQ, UK; Research Data Team, The Turing Institute, 96 Euston Rd, London NW1 2DB, UK; European Bioinformatics Institute, European Molecular Biology Laboratory, Welcome Genome Campus, Hinxton CB10 1SD, UK

## Abstract

The International Mouse Phenotyping Consortium (IMPC; https://www.mousephenotype.org/) web portal makes available curated, integrated and analysed knockout mouse phenotyping data generated by the IMPC project consisting of 85M data points and over 95,000 statistically significant phenotype hits mapped to human diseases. The IMPC portal delivers a substantial reference dataset that supports the enrichment of various domain-specific projects and databases, as well as the wider research and clinical community, where the IMPC genotype–phenotype knowledge contributes to the molecular diagnosis of patients affected by rare disorders. Data from 9,000 mouse lines and 750 000 images provides vital resources enabling the interpretation of the ignorome, and advancing our knowledge on mammalian gene function and the mechanisms underlying phenotypes associated with human diseases. The resource is widely integrated and the lines have been used in over 4,600 publications indicating the value of the data and the materials.

## INTRODUCTION

The International Mouse Phenotyping Consortium (IMPC; https://www.mousephenotype.org/) produces whole gene knock out (KO) mouse lines for community use and delivers broad comprehensive phenotyping data and the KO lines as an international resource enabling functional analyses with genome-wide coverage as the eventual goal (1). The lines and the data are vital resources for elucidating both the genetics and the mechanisms underlying phenotypes associated with human diseases and they inform our understanding of the ‘ignorome’—the portion of the mammalian coding genome which is poorly functionally characterised and which we seek to illuminate.

Since its inception in 2011, IMPC has published 17 data releases describing over 8000 genes and their associations to 90 000 significant phenotypes, in addition to 750 000 images and over 1000 human disease models. Standardisation and use of established ontologies underpin the integration and publishing of the resource. The International Mouse Phenotyping Resource of Standardised Screens (IMPReSS) database drives the validation of the raw data, while the Mammalian Phenotype Ontology (MP) (2) is used to capture mammalian phenotypes and to enable correlation to human phenotypes defined by the Human Phenotype Ontology (3). The knowledge externalised by the resource supports the identification of new mouse models of rare and common human diseases, new gene functions and the development of novel methodological approaches that form the basis of new gene–disease associations. Moreover, it underpins successful experiments effectively uncovering pleiotropy (4–6), of particular importance when elucidating the genetic causes of syndromic disorders, as well as wide-ranging sexual dimorphism (7).

The IMPC data exists in a complex ecosystem of projects and databases that are evolving to deliver systematic analyses of cellular, organism level and population analyses. All of these contribute to our understanding of function and will extend beyond coding regions to deliver a map of genotype–phenotype understanding. IMPC delivers data for integration with resources such as the Mouse Genome Database (8) or the Monarch Initiative (9), and clinical resources enabling the analysis of rare disease patient genomes (10).

Here, we provide an overview of the latest data release and of the IMPC Web Portal, we discuss a series of use cases and before concluding, we present a brief description of the complex IMPC data flow.

## THE IMPC RESOURCE

Data Releases (DR) provide a stable and versioned reference set of analysed IMPC data covering all data generated by the consortium until the release date - or alternatively, all data incorporated in a previous data release augmented with the increment generated since that release. These timestamped releases are critical for reproducible analyses and enable a clear provenance-based integration into external resources. Newly generated data between releases (by the production centres) is collected, stringently QCed and run through an Extract-Transform-Load, statistical and the disease association pipelines before being integrated with the current release and packaged into a new release.

**Figure 1.**
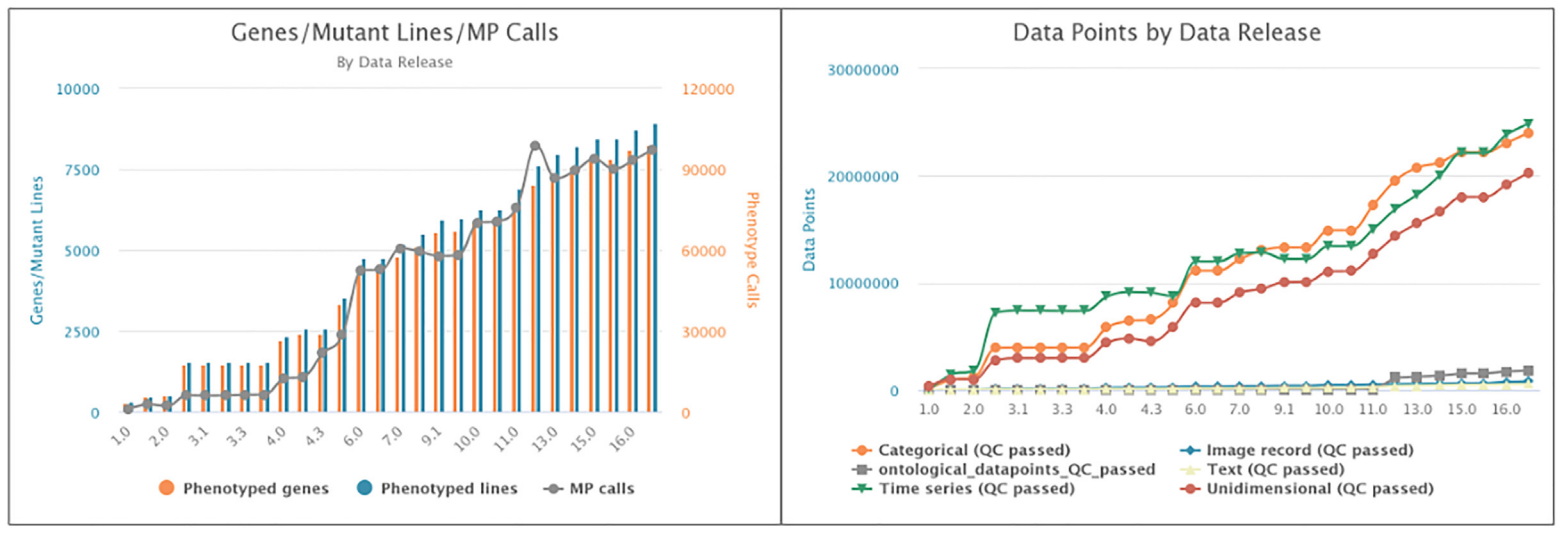
IMPC data releases evolution over the last 11 years.

IMPC publishes three data releases per year. Figure 1 depicts the evolution of the number of knockouts and number of data points across the 11 years of IMPC existence. FAIRness is ensured by using standard identifiers—MGI IDs—and community ontologies—for example, MP and HPO. Table 1 lists the numbers associated with the latest release—DR17 from 19 July 2022. DR17 covers a total of 85 911 461 data points on 8267 genes and 97 294 significant phenotypes aligned to 1321 human diseases. In addition, it also includes 786 081 images with a total size of ∼1.6TB, generated from 21 procedures spanning all life stages. For example, gene Cib2 (https://www.mousephenotype.org/data/genes/MGI:1929293) is associated with 12 significant phenotypes, inferred from 241 measurements and 424 data points, and enriched with 162 images (79 echo, 65 X-rays, 8 LacZ embryo, 7 LacZ adult and 3 ABR waveforms).

**Table 1.** IMPC Data Release 17 (DR17; 19 July 2022) statistics covering genes, associated phenotypes and the procedures supporting the data collection and QC

Genes	8267
Lines	8916
Significant phenotypes	97 294
Total data points	85 911 461
Ontology annotations	1 805 447
Images	786 081
Human disease models	1321
Total number of procedures	80
Total number of parameters	5084
Metadata parameters	306
Parameters analysed for phenotypes	552
Media parameters	66

To ensure data consistency, integrity and shareability, the IMPC phenotyping protocols are standardised and are available on IMPReSS. DR17 was supported by an IMPReSS version containing 80 procedures with 5084 parameters. Of these parameters 1164 record metadata, 2019 are analysed for phenotypes and 66 are media parameters (more details are provided in the Data Flow section).

**Figure 2.**
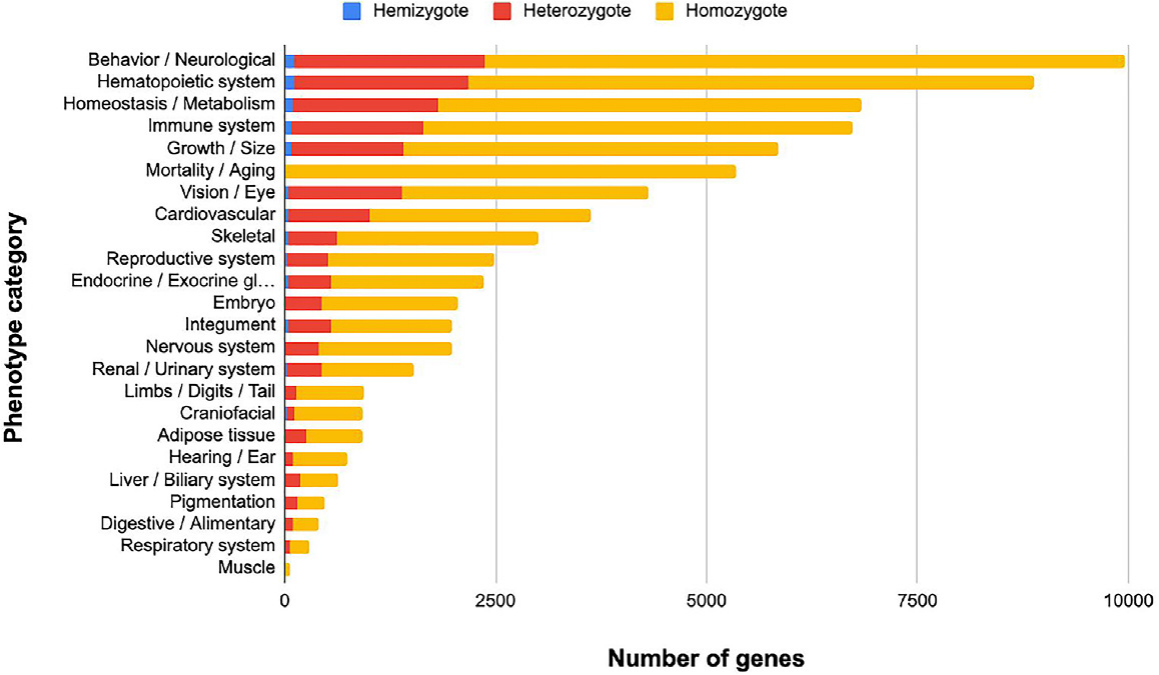
IMPC Data release 17 (DR17; 19 July 2022) phenotype categories.

Finally, Figure 2 depicts the number of knockouts split across phenotype categories, with the top being *behavioural* phenotypes and phenotypes of the *hematopoietic system*.

All data collected by the IMPC is freely available and several access channels are provided, based on the individual needs of the users - ranging from individual items to complete data releases. Programmatic access to the entire range of up-to-date IMPC data is available through an Application Programming Interface (API), documented at https://www.mousephenotype.org/help/programmatic-data-access/. Snapshots of the entire dataset captured at the time of each data release are also available for bulk download via FTP (ftp://ftp.ebi.ac.uk/pub/databases/impc/).

## THE IMPC WEB PORTAL

**Figure 3.**
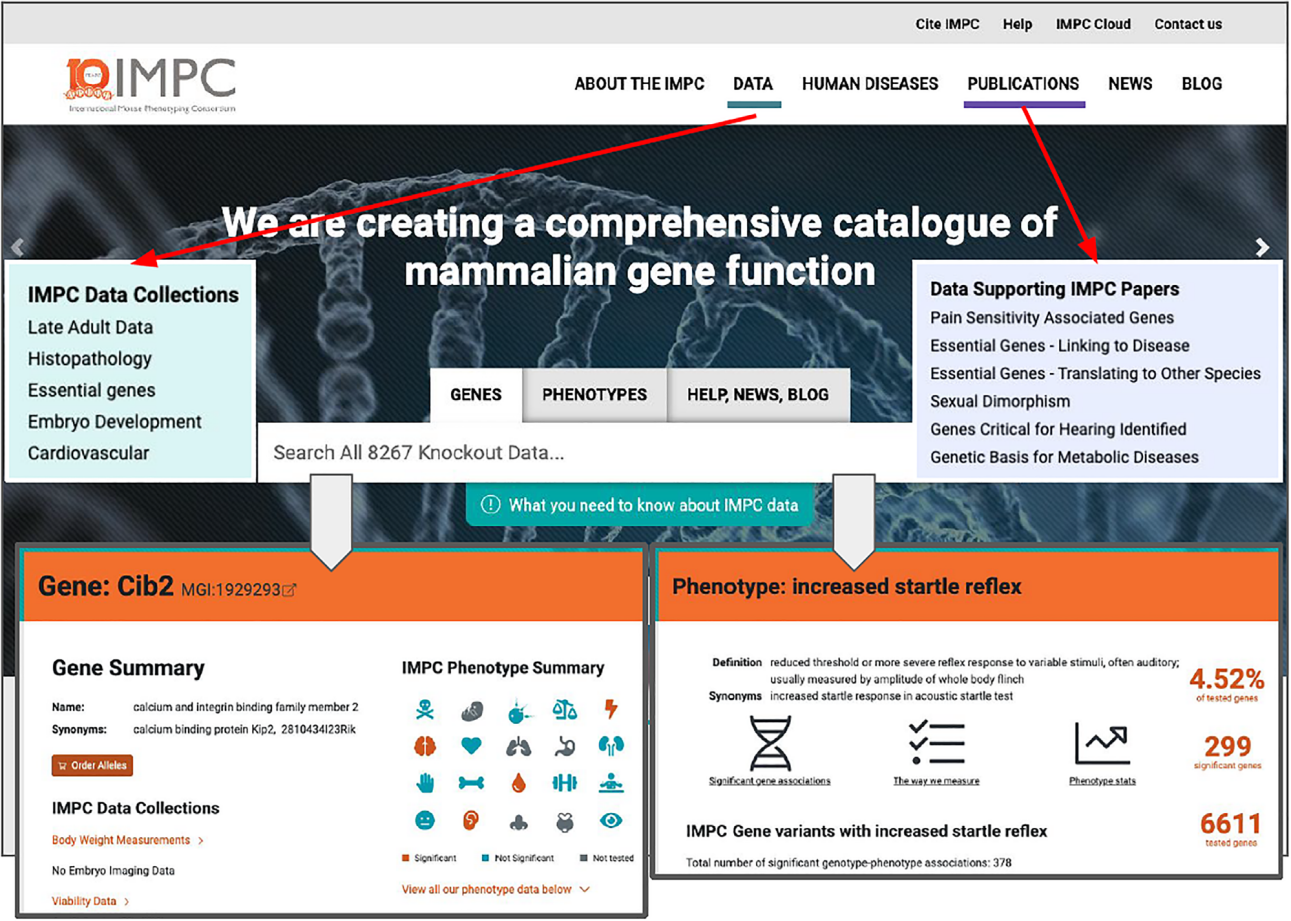
The IMPC Web portal. The main page provides immediate access to gene and phenotype-based queries, domain-specific data collections, data supporting IMPC publications and help and training materials

The IMPC Web Portal enables users to access and explore the resource in a simplified format. In 2019, after engaging various user groups, including clinicians and biomedical researchers, the Web Portal underwent a major redesign, which led to a new data access and browsing paradigm focusing on common user journeys and simpler entry points for new users. To improve findability, the top-level menu provides immediate access to both data as well as impact, via publications showcasing the use of IMPC data (Figure 3). The ‘Data’ section, in addition to advanced visualisation tools and options for data download, also includes bespoke data collections. These organise data into biological themes to provide easy entry points for users searching for cardiovascular, embryo development, histopathology, essential genes and late adult data. Similarly, the ‘Publications’ section enables users to immediately access literature demonstrating the impact of and knowledge generated with IMPC data.

The home page of the Web Portal allows users to explore the IMPC knowledge using names or identifiers for phenotypes and genes - as shown in Figure 3. The key information in IMPC is encoded using widely-adopted nomenclatures and ontologies. This strategy assists users searching for phenotypes using a synonym or for genes using MGI IDs, mouse or human symbols.

**Figure 4.**
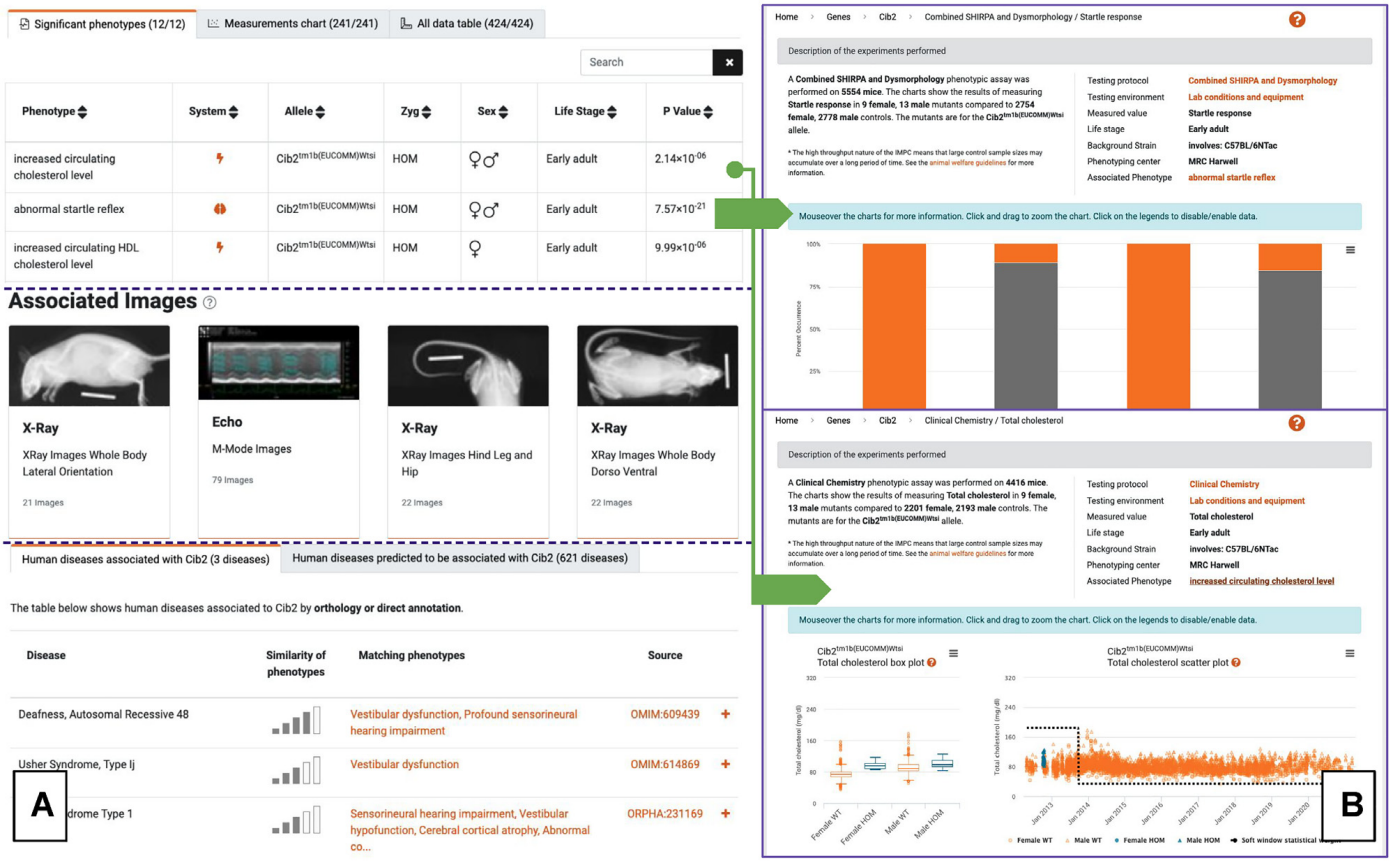
The IMPC gene and phenotype pages exemplified using the Cib2 gene and specific details on the phenotypes increased circulating cholesterol level and abnormal startle reflex. (**A**) Gene pages display data and information using a block-oriented approach, with each block focusing on one aspect—for example, significant phenotypes, images, publications. (**B**) Phenotype pages provide in-depth visual analyses and comprehensive provenance information.

Gene pages are the central component of the IMPC Web Portal, displaying the corresponding KO lines, physiological systems affected (e.g. *cardiovascular*, *nervous*, *hematopoietic*, etc.—see Figure 3), the significant phenotypes detected (e.g. *abnormal bone structure*, *increased grip strength*, etc), images available, publications using these lines and relationships to human disease (Figure 4, panel A). Moreover, they provide an entry point into accessing all the data and statistical analyses underpinning the phenotype calls represented as chart pages, which display advanced visualisations over the data (Figure 4, panel B), comprehensive descriptions of both the assays and the statistical analyses performed and a complete provenance trail. Finally, links to external sources, such as, MGI or the Monarch Initiative are provided in appropriate contexts.

The Documentation (Help) section of the Web Portal covers always the most up-to-date IMPC content and is iteratively updated to reflect changes in the way we present results or in our statistical analysis practices. In addition, the ‘Getting Started with IMPC Data’ section links to a comprehensive series of training materials and to information on gaining access and using the resource.

## USE CASES

IMPC focuses on delivering a reference dataset that enables functional analyses using the mammalian genome and hence supporting the enrichment of various domain-specific downstream projects and databases. These range from the mouse genome database, where IMPC contributes with mouse alleles and phenotypes, to the wider research and clinical community, where IMPC genotype–phenotype knowledge contributes to the genetic diagnosis of patients as well as various integration projects.

The resource adds value to the data through processes that efficiently annotate, integrate and disseminate results in context and via different modalities helpful to our stakeholders listed in Table 2. These efforts, primarily communicated via the Web Portal and scientific papers, provide the research community with new mouse models for further investigation (e.g. deeper characterization of a particular physiological system) and thousands of gene function predictions at scale. These allow the generation of new hypotheses that are increasing our understanding of the mammalian genome, forming the basis of new research, and identifying disease associations. Table 2 showcases the impact of the resource via advances and contributions to the generation of new knowledge that has been possible with the emergence and analysis of IMPC data.

**Table 2.** IMPC data stakeholders, associated use cases and impact

IMPC data stakeholders	Use case	New knowledge
Biomedical researchers	Access statistically significant phenotypic associations for a given gene	Demonstrated sexual dimorphism (7); identification and characterization of essential genes for embryonic development (13) identification of genes associated with hearing loss (14), metabolic processes (15,16), eye development (17), bone mineral density (18), congenital and structural heart disorders and cardiomyopathy (19), pain sensitivity (20)
Rare disease researchers	Search for specific phenotypes or genes of interest	Translational pipeline using the HPO-annotated clinical descriptions of patients and MPO-annotated IMPC mice, allowing to establish gene–disease associations or models (21,22)
Common disease researchers	Search for functional characterisation of genes with association to disease derived from GWAS or effector gene analyses	Systematic detection of co-morbidities, facilitating the association of genes with human diseases and pleiotropy (4–6)
Essential genes researchers	Gather evidence from the IMPC viability pipeline, including embryonic screening and imaging data	Identification and characterization of genes that are essential for organism viability, critical for development and health (12,13,23)
Researchers, in general	Search for mouse lines or samples with phenotype data available, e.g., someone wishing to conduct secondary targeted phenotyping experiments on well-known genes to augment existing broad-based phenotype data	Deeper phenotypic characterization of IMPC mice, covering bone, immunophenotyping, brain morphogenesis, hearing loss, pulmonary metastatic colonisation (24–30)
Data scientists	Seek access to large-scale standardised gene-phenotype datasets to perform their own analyses in combination with other datasets	Application of pipeline using HPO-encoded clinical descriptions and MPO-encoded mouse KO descriptions to orthologue mapping, essential gene classification system, automated image analyses and statistical analysis of long-term series (22,23,31–34)
Informatics users	Access all, or partial, datasets for inclusion in their own resource set	Monarch Initiative (9); Illuminating the Druggable Genome (35); OpenTargets (36)
Funding bodies and programme staff	Track the progress of mouse production and phenotyping efforts, and the state of production, collection and dissemination of the data	Insights into genome editing techniques using ES cells or CRISPR/Cas9 genome editing methods; relevance for clinical genome editing (37–40)

Notable examples demonstrating immediate clinical utility include the analyses on Tanc2 or Tmem63b. The former provided support for diagnosis of 20 patients with a neurodevelopmental disorder syndrome with rare de novo and inherited disruptive mutations in TANC2 (11). While a subset of patients displayed hyperactivity, the challenge was the lack of a detailed understanding of the pleiotropic effects of the mutations. The Tanc2 IMPC mouse knockouts (homozygous viable) also displayed hyperactivity, in addition to a reduced body size and adipose tissue and an altered metabolic rate. More importantly, their phenotype included liver abnormal cellular morphology and dysfunction (abnormal metabolite levels), which in humans are easily accessible via biomarkers of liver dysfunction. This enabled the detection of an abnormal liver function, which may lead to systemic disease (diabetes/arteriosclerosis/cardiovascular disease/stroke). Similarly, hyperactivity and limb-grasping phenotypes recorded on IMPC Tmem63b knockouts (homozygous lethal phenotype, heterozygous viable) were used to connect five patients with three variants (one overlapping in three patients) (12).

A major use case of the IMPC data are human disease models. These serve a dual purpose: (i) to evaluate the recapitulation of human phenotypes for known gene–disease associations in the mouse knockout and (ii) to uncover potential novel gene–phenotype associations by identifying mouse knockouts mimicking clinical features observed in patients. These phenotypic similarity computations between mouse knockouts and known human disorders are performed by the PhenoDigm algorithm (34), which relies on the availability of standardised phenotypic annotations (2,3). The former objective is illustrated in Table 3, that summarises the number of IMPC mouse knockouts with a one-to-one human orthologue associated with OMIM /Orphanet disorders (41,42) and the corresponding PhenoDigm results according to DR17. With regard to predicted gene–disease associations for genes not known to be associated with Mendelian disorders, the evidence provided by these similarity computations successfully contributed to identifying novel developmental disorder genes (22). Human disorders associated with a gene by orthology (i) and human disorders predicted to be associated with a gene based on PhenoDigm scores (ii) are available in each Gene Page.

**Table 3.** Mouse orthologues of human Mendelian disease-associated genes that entered the IMPC phenotyping pipeline according to DR17.0 and potential models for these disorders

Mouse genes associated with a Mendelian disorder by orthology that have entered the IMPC phenotyping pipeline	2574
Genes with MP (mouse knockout) and HPO (disorder) annotations available to compute a phenotypic similarity score by PhenoDigm	2223
Genes with a PhenoDigm match (automated recapitulation of disorder phenotypes)	1155
Genes associated preweaning lethality in the homozygous knockout and with reports of prenatal to childhood death in humans (manual curation, not captured by the algorithm)	166

## THE IMPC DATA FLOW

**Figure 5.**
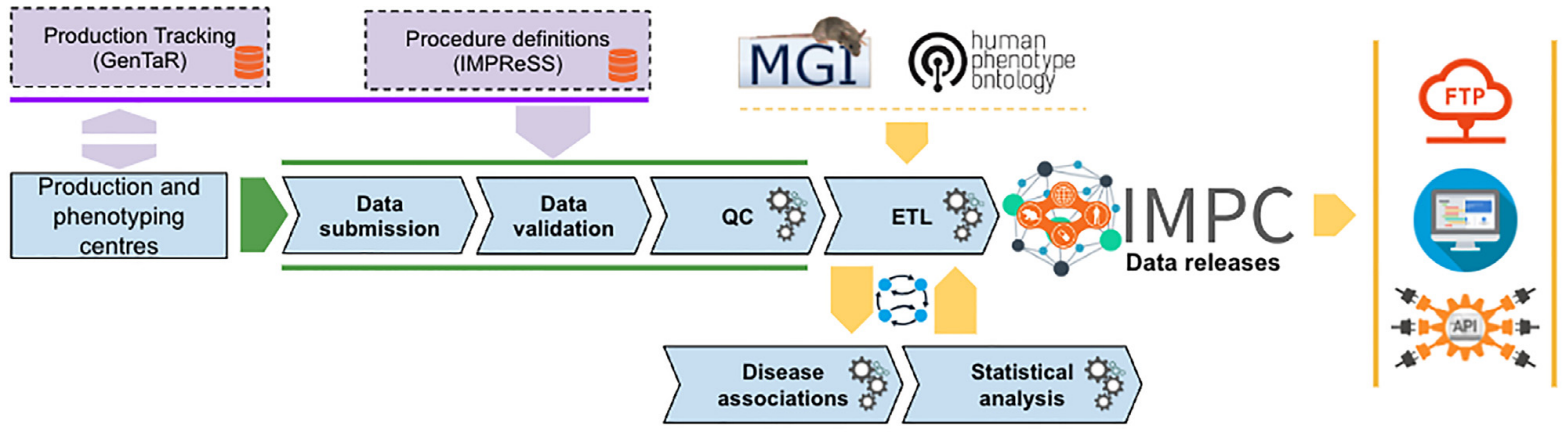
The IMPC data flow. Data generated by the phenotyping centres via the systematic phenotyping pipeline is aggregated, validated and undergoes stringent QC procedures before being served as input to the Extract-Transform-Load and statistical analysis pipeline. The outcome—a curated genotype–phenotype resource - is made available via data releases and the IMPC Web Portal.

The IMPC strives to generate and deliver open, quality controlled, robust and reproducible data to the scientific and user community. Figure 5 depicts the flow of the data within the overall IMPC ecosystem from mouse production to curated data made available via packaged releases or the Web Portal. The starting point is the coordination between the mouse production facilities.

Tracking the status of mouse production is a critical activity that underpins the fundamental aim of the program to maximise the number of genes knocked out and minimise the number of animals. A tracking system—GenTaR (https://www.gentar.org/tracker/#/) is used to minimise overlap, enable QC of data and to provide progress updates to external researchers who registered their interest on specific genes.

Raw data produced by the centres is crawled on a daily basis and validated against the definitions captured by IMPReSS. This ensures data is consistent across all centres. As the consortium’s and the wider scientific community’s data analysis efforts have matured, the role of high-quality data has been key to ensure reproducibility and an appropriate foundation for hypothesis testing. IMPReSS is designed in collaboration with the phenotyping centres and the scientific community and specifies (in human and machine readable formats) both how the procedures are to be performed and how and what data is to be submitted by the phenotyping centres.

**Figure 6.**
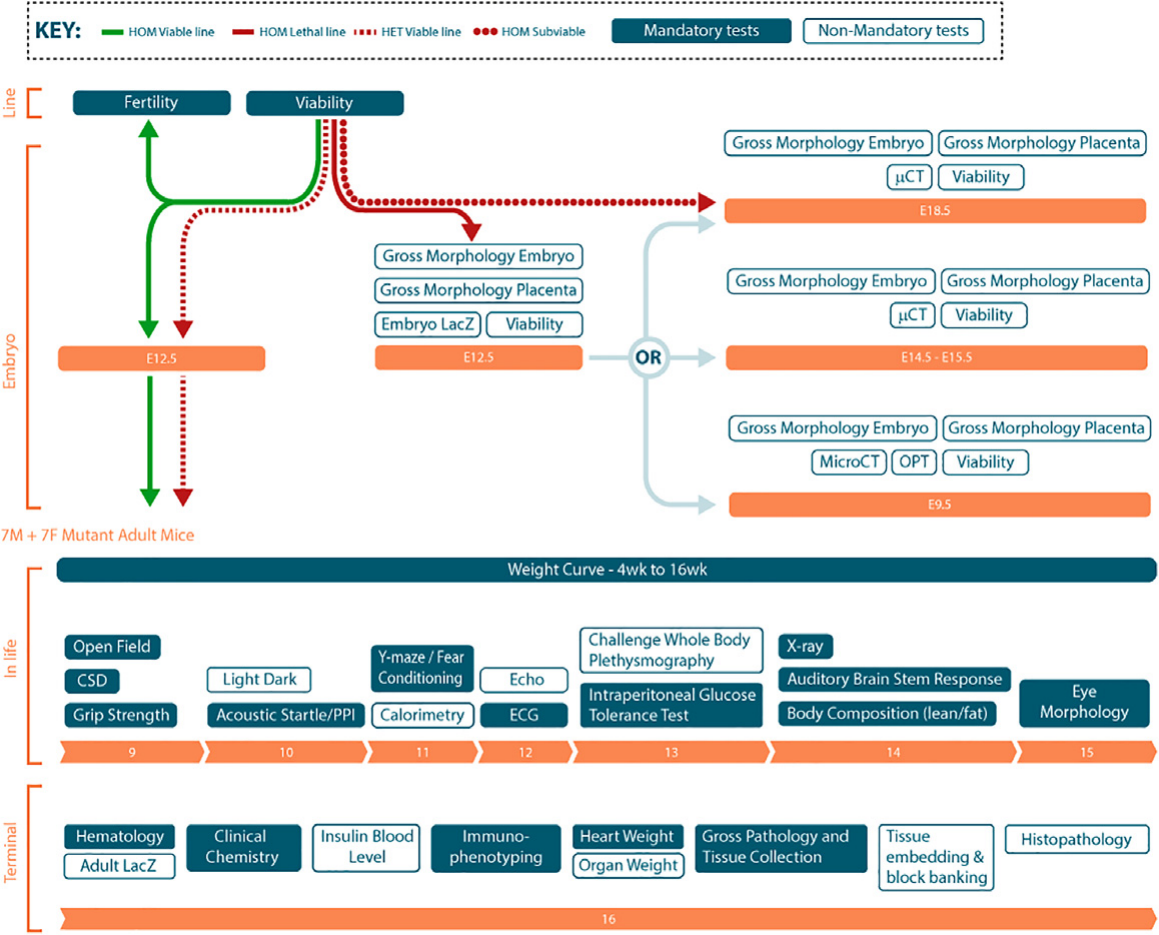
The IMPC phenotyping pipeline.

The procedures are organised in pipelines, which define the order and age at which the procedures are to be performed. The core IMPC Early Adult (EA) in vivo pipeline starts at 9 weeks running to 16 weeks with behavioural, morphological, metabolic and cardiovascular assessments (Figure 6). Viability, fertility and embryo procedures are also carried out. Additional centre specific pipelines allow inclusion of procedures not part of the core pipeline. With the extension of the project to later adult phenotyping, Late Adult (LA) and Interval pipelines are also defined on IMPReSS. The LA pipelines are based on the core EA pipeline with some procedures removed due to biological changes in the baseline animals, such as deafness, which would not result in meaningful results. Each procedure defines the parameters to collect data for, including the metadata parameters, and the specifications for them.

All the data goes through a stringent QC process. The QC checks include identifying high, low and impossible values, inconsistencies between values for different parameters, data shifts and drifts and date QC. As an example, all data for parameters in Open Field are assessed for high and low values and data drifts and shifts over time. As the parameters record data for different sections of the experimentation arena, in a number of time periods and the totals, the consistency of these relationships is also assessed; for example the sum of periphery and centre distance travelled should match the total distance. The date of experiment will be checked against the specimen date of birth to confirm the procedure was performed at the right age.

Data that passes these quality checks, in addition to the associated imagery, is provided as input to an Extract, Transform and Load (ETL) pipeline that prepares it for packaging and publishing via versioned data releases. The ETL pipeline consolidates the integration of cross-reference data from internal and external sources—e.g., GenTaR, the Mouse Genome Database (8) and various ontologies - and standardises the production of datasets for ingestion by the statistical and the disease association pipelines.

To identify pheno-deviant lines, data generated from knockout mice are subjected to statistical analysis where the parameters measured during the assay are compared with the same parameters measured in parallel from control wild-type mice from an identical background strain. When a test is considered statistically significant, ontology terms from the Mammalian Phenotype Ontology (MP) (2) are automatically associated to the individual genotypes based on association specified in IMPReSS for every analysed parameter. Subsequently, the disease association pipeline—developed in collaboration with the Monarch Initiative (9)—generates a score for how phenotypically similar an IMPC line is to a particular human disease. This is achieved based on the similarity of terms in the Human Phenotype Ontology (3) used to annotate rare diseases in humans and MP terms used to annotate the IMPC procedures.

The final step of the data flow is the packaging of the data into versioned bundles and making it available for users on the Web Portal, programmatically via APIs and as downloadable archives on FTP.

## CONCLUSION

The IMPC focuses on delivering a reference dataset consisting of comprehensive and standardised phenotyping data that enables functional analyses using the mammalian genome. We will continue to deliver a robust, high quality and reproducible dataset, maximally integrated within the wider knowledge ecosystem focused on advances to human health.

For the future, we aim to bring more depth to the resource by integrating additional, relevant knowledge sources to enable novel analyses. For example, as complex disease analyses move from association studies to causative or ‘effector’ gene analyses, the functional data provided by IMPC and others is critical to understand the phenotypic consequences of variants, to understand which genes are essential and which contribute to diseases enabling downstream functional follow up.

The IMPC phenotype descriptions listed in the context of genes in the Ensembl genome browser, MGI or the OpenTargets platform are examples of a successful outgoing data integration. A similar, untapped resource is the UCSC Genome Browser. While a phenotype and literature track exist in the context of the human genome (covering data sources such as OMIM), this is absent in the context of the mouse genome. We aim to fill this gap and enable IMPC to be the premier mouse phenotype data provider for the UCSC Genome Browser.

Finally, advanced visualisation capabilities will be added to the Web Portal to improve the user experience and the exploratory discovery and interpretation of the data. Heatmaps have been implemented on the ‘Late adult data’ page to enable users to inspect the genes that have been phenotyped for the ageing procedures. We will develop similar widgets on all other Data collection pages, including the automated body system pages. We will also add gene clustering options based on phenotype (MP) and Gene Ontology (GO) component classes to enable an understanding of the relationship between the impacted biological processes and the expressed phenotype.

## DATA AVAILABILITY

- IMPC Web Portal: https://www.mousephenotype.org/
- IMPC FTP data releases: http://ftp.ebi.ac.uk/pub/databases/impc/
- The Mammalian Phenotype Ontology: https://github.com/mgijax/mammalian-phenotype-ontology
- The Human Phenotype Ontology and its resources: http://www.human-phenotype-ontology.org
- UPheno: https://github.com/obophenotype/upheno
